# Correction: Oh, J.Y., et al. Synergistic Autophagy Effect of miR-212-3p in Zoledronic Acid-Treated In Vitro and Orthotopic In Vivo Models and in Patient-Derived Osteosarcoma Cells, *Cancers* 2019, *11*, 1812

**DOI:** 10.3390/cancers12061359

**Published:** 2020-05-26

**Authors:** Ju Yeon Oh, Eun Ho Kim, Yeon-Joo Lee, Sei Sai, Sun Ha Lim, Jang Woo Park, Hye Kyung Chung, Joon Kim, Guillaume Vares, Akihisa Takahashi, Youn Kyoung Jeong, Mi-Sook Kim, Chang-Bae Kong

**Affiliations:** 1 Laboratory of Biochemistry, Division of Life Sciences, Korea University, Seongbuk-gu, Seoul 02841, Korea; ojo5295@kirams.re.kr (J.Y.O.); joonkim@korea.ac.kr (J.K.); 2 Division of Radiological Science and Clinical Translational Research, Korea Cancer Center Hospital, Nowon-gu, Seoul 01812, Korea; 3 Department of Biochemistry, School of Medicine, Daegu Catholic University, Nam-gu, Daegu 42472, Korea; eh140149@cu.ac.kr (E.H.K.); sunha112@cu.ac.kr (S.H.L.); 4 Division of Radiation Biomedical Research, Korea Institute of Radiological and Medical Sciences, Seoul 01812, Korea; eyeonjoo@gmail.com; 5 Department of Basic Medical Sciences for Radiation Damages, National Institute of Radiological Sciences, Chiba 263-8555, Japan; sai.sei@qst.go.jp; 6 Korea Drug Development Platform Using Radio-Isotope, Korea Institute of Radiological & Medical Sciences, Seoul 139-706, Korea; jangwoo@kirams.re.kr (J.W.P.); hkchung@kirams.re.kr (H.K.C.); 7 Cell Signal Unit, Okinawa Institute of Science and Technology Graduate University (OIST), Okinawa 1919-1, Japan; guillaume.vares@oist.jp; 8 Gunma University Heavy Ion Medical Center, Maebashi 371-8511, Gunma, Japan; a-takahashi@gunma-u.ac.jp; 9 Research Center for Radiotherapy, Korea Institute of Radiological and Medical Sciences, Seoul 139-706, Korea; amy3523@kirams.re.kr; 10 Department of Radiation Oncology, Korea Institute of Radiological and Medical Sciences, Seoul 139-706, Korea; 11 Department of Orthopaedic Surgery, Korea Institute of Radiological and Medical Sciences, Seoul 139-706, Korea

The authors wish to make the following corrections to this paper [1]:

The authors would like to replace Figure 3b in [1]. The correction is to correct errors in the arrangement of the Western blots. The original version of Figure 3b is:

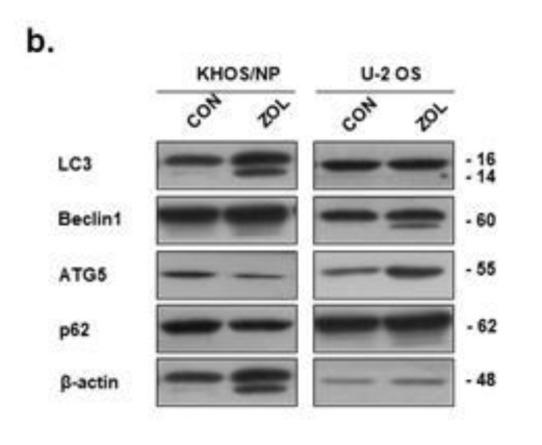



and should be replaced with the following Figure 3b:

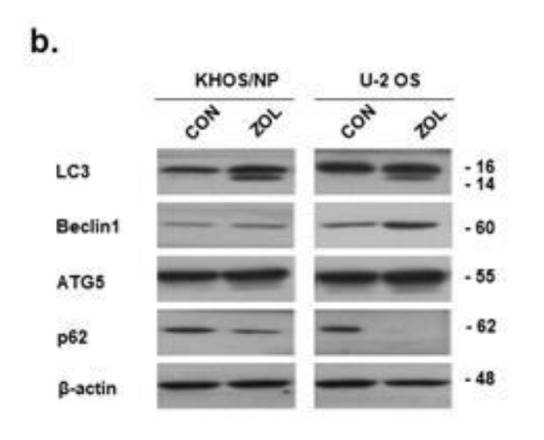



The authors would like to replace the first affiliation in [1]. The original affiliation is “Laboratory of Biochemistry, School of Life Sciences and Biotechnology, Korea University, Seongbuk-gu, Seoul 136-701, Korea” and should be replaced with “Laboratory of Biochemistry, Division of Life Sciences, Korea University, Seongbuk-gu, Seoul 02841, Korea”.

The authors would like to apologize for any inconvenience caused to the readers by these changes.
