# Supplementary material for: Synergistic Autophagy Effect of miR-212-3p in Zoledronic Acid-Treated In Vitro and Orthotopic In Vivo Models and in Patient-Derived Osteosarcoma Cells
Source: Cancers (Basel). 2019 Nov 18;11(11):1812. doi: 10.3390/cancers11111812 (PMC6895802; doi:10.3390/cancers11111812)
Supplement: Supplementary file 1 [file cancers-11-01812-s001.pdf]

Supplementary Materials

Synergistic Autophagy Effect of miR-212-3p in Zoledronic Acid-treated in Vitro and Orthotopic in Vivo Models and in Patient-derived Osteosarcoma Cells

Ju Yeon Oh, Eun Ho Kim, Yeon-Joo Lee, Sei Sai, Sun Ha Lim, Jang Woo Park, Hye Kyung Chung, Joon Kim, Guillaume Vares, Akihisa Takahashi, Youn Kyoung Jeong, Mi-Sook Kim and Chang-Bae Kong

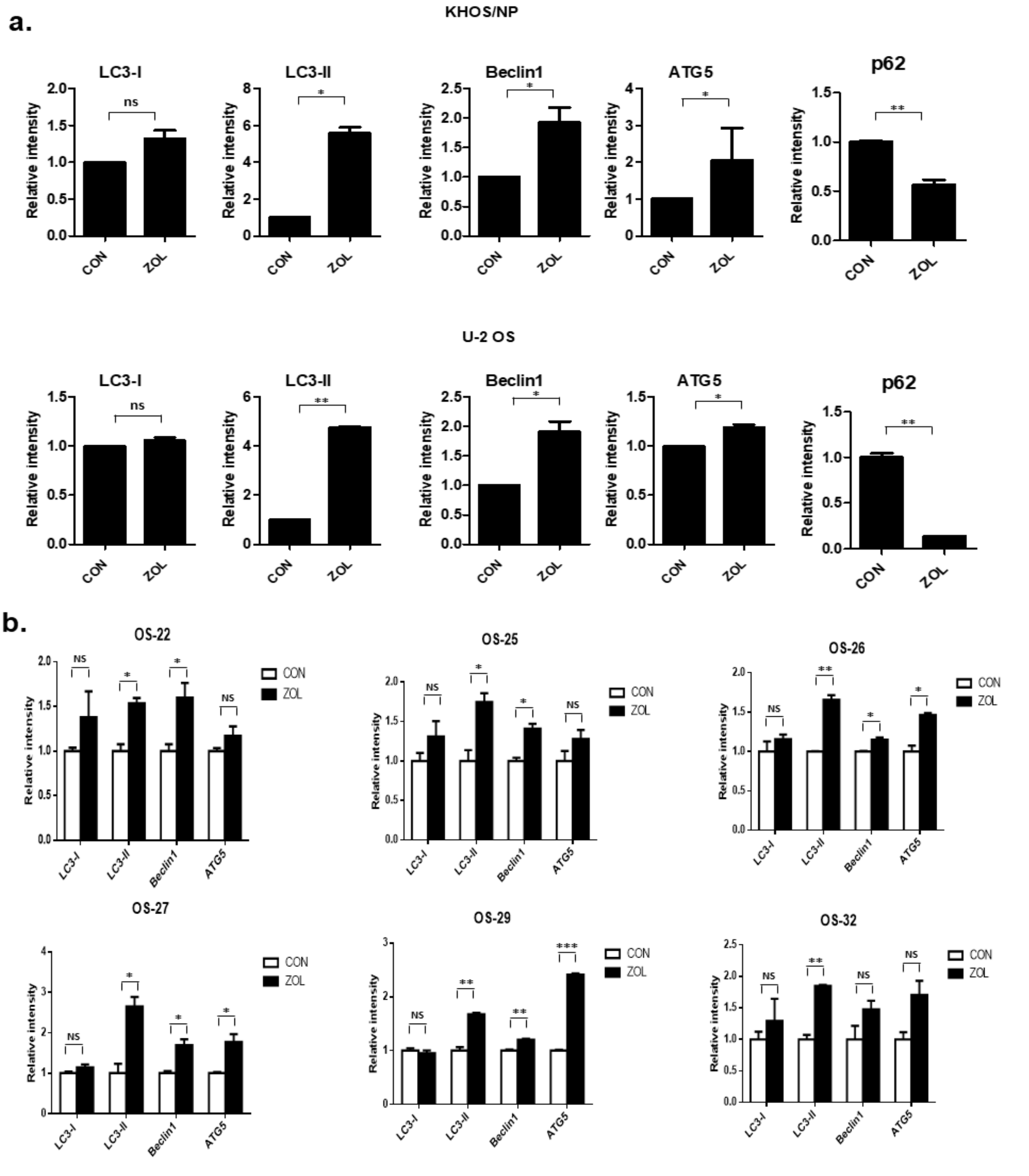

Figure S1. Band intensities for target proteins were normalized to that for β-actin. (**a**,**b**) Band intensities for target proteins were normalized to that for β-actin. Values represent the means of 3 experiments ± SD; **p* < 0.05, ***p* < 0.001.

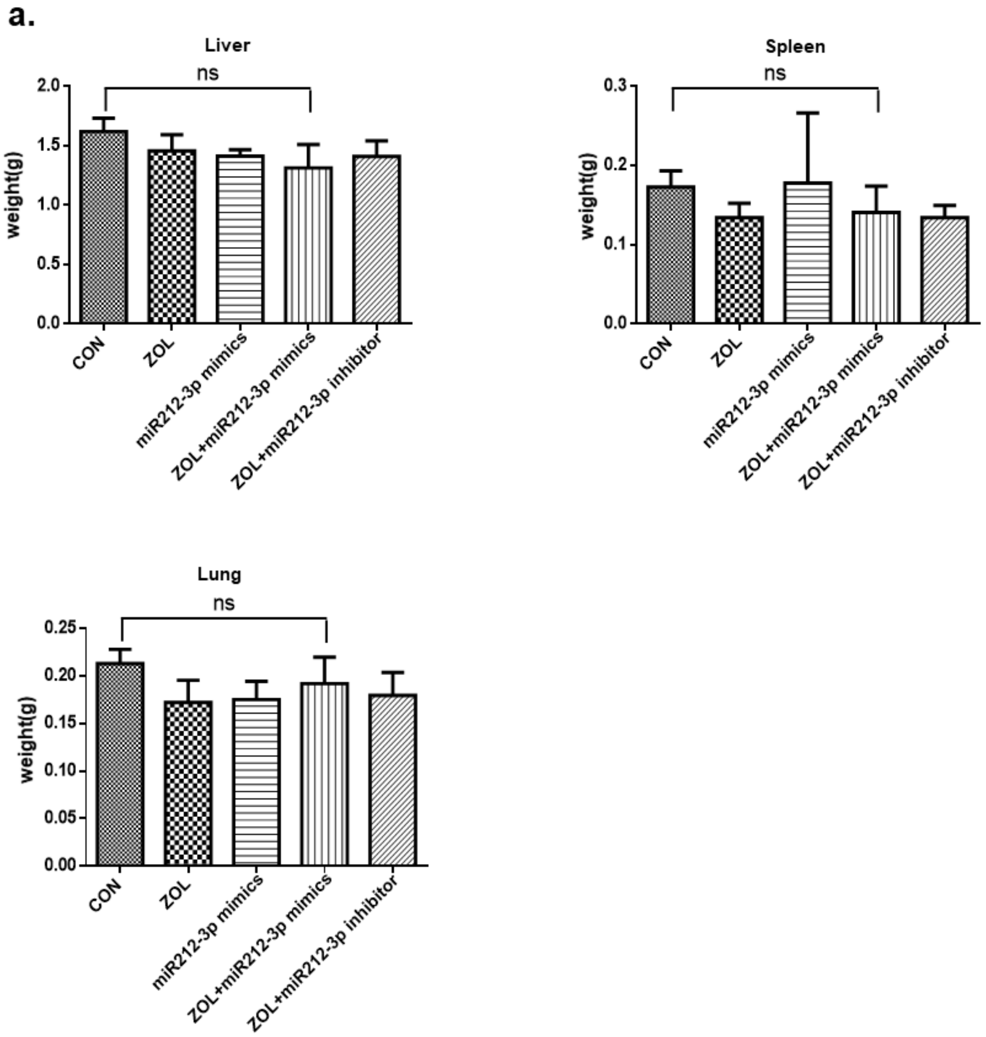

Figure S2. Spleen, liver and lung of mice were excised and weighed. (**a**) Spleen, liver and lung of mice were excised and weighed at the end of the experiment (28 days) for control, ZOL, miR212-3p mimics, ZOL+ miR212 mimics, and ZOL+ miR212 inhibitor; ns > 0.05.

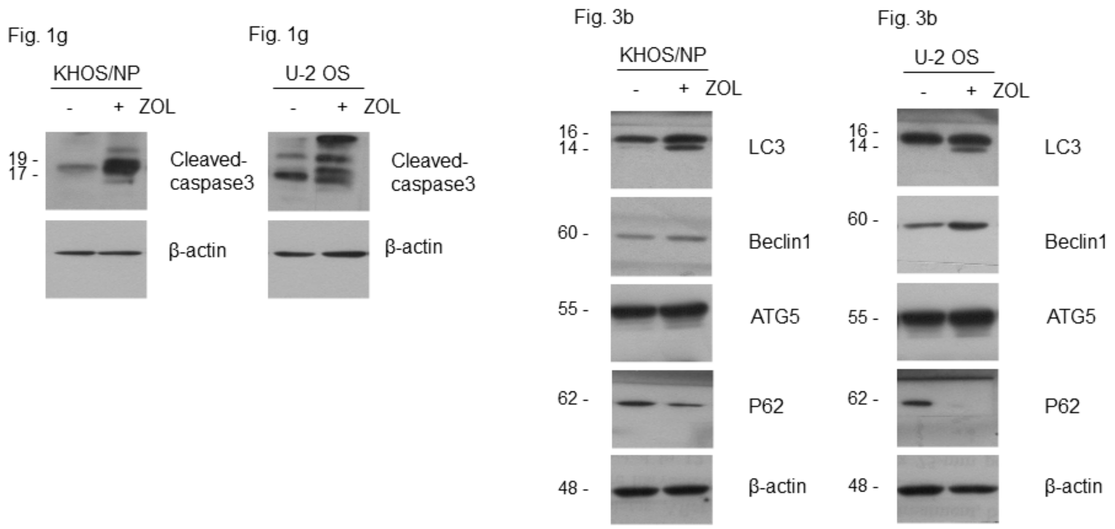

Fig. 3d

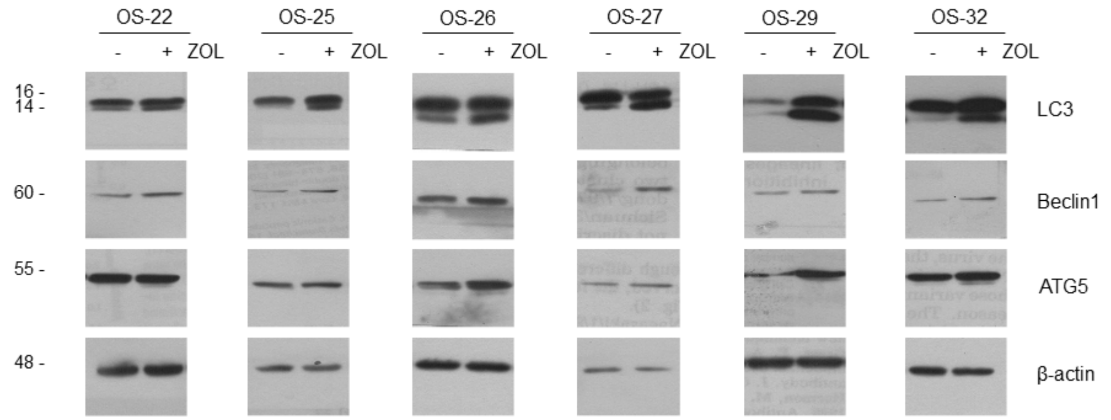

Fig. 4a

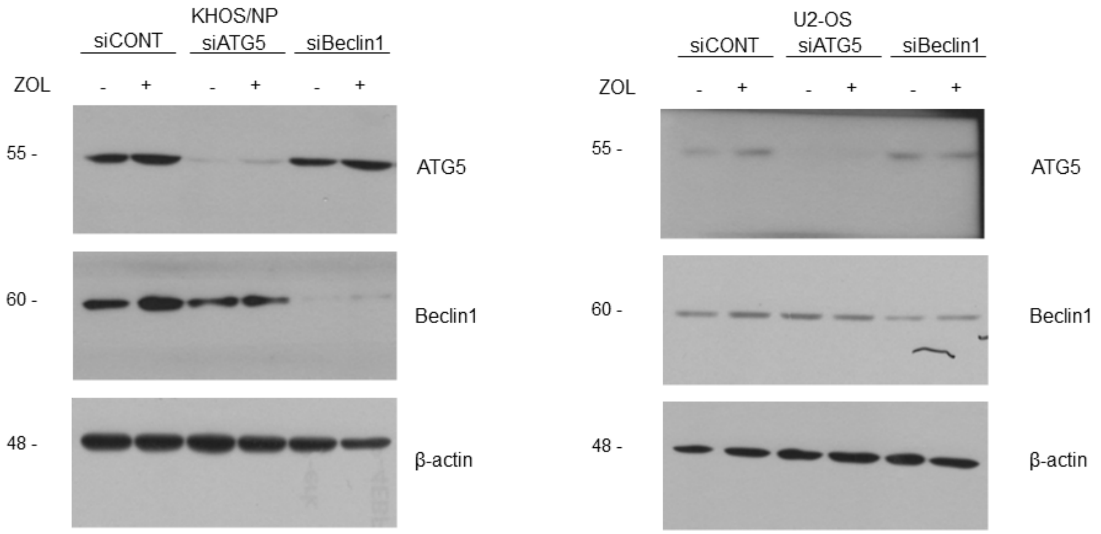

Fig. 5a

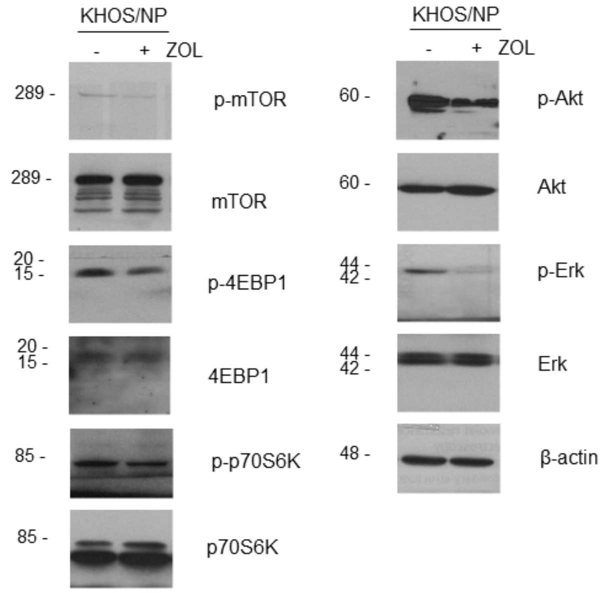

Fig. 5a

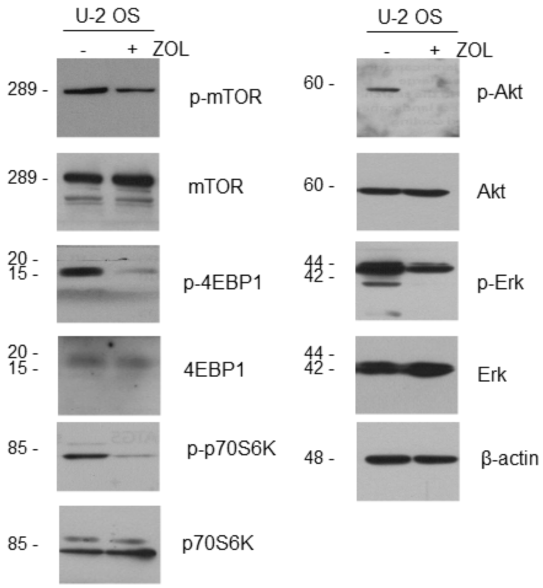

Fig. 6h

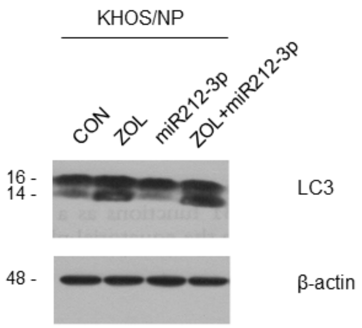

Fig. 6h

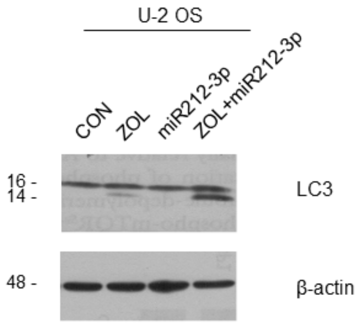

© 2019 by the authors. Licensee MDPI, Basel, Switzerland. This article is an open access article distributed under the terms and conditions of the Creative Commons Attribution (CC BY) license (http://creativecommons.org/licenses/by/4.0/).
